# Problematic Smartphone Use and Occupational Balance Among High School Students: A Cross-Sectional Study

**DOI:** 10.3390/bs16101855

**Published:** 2026-10-09

**Authors:** Selin Davun, Mehmet Akif Sezerol, Murat Tuğberk Bakar, Melek Nur Aslan, Şeyma Halaç

**Affiliations:** 1 Sultanbeyli District Health Directorate, Istanbul 34930, Türkiye; murattugberk.bakar@saglik.gov.tr; 2 Department of Public Health, Hamidiye Faculty of Medicine, University of Health Sciences, Istanbul 34668, Türkiye; mehmetakif.sezerol@sbu.edu.tr; 3 Epidemiology Program, Institute of Health Sciences, Istanbul Medipol University, Istanbul 34810, Türkiye; 4 Fatih District Health Directorate, Istanbul 34091, Türkiye; meleknur.aslan@saglik.gov.tr; 5 General Directorate of Health Promotion, Ankara 06800, Türkiye; seyma.halac@saglik.gov.tr

**Keywords:** adolescents, problematic smartphone use, occupational balance, Adolescent Occupational Balance Scale, sleep, digital behavior

## Abstract

Problematic smartphone use (PSU) may affect how adolescents organize and experience everyday occupations, yet evidence on occupational balance in school populations is limited. This cross-sectional study examined the association between PSU and occupational balance among 2681 students from six public high schools in Istanbul, Türkiye. PSU was assessed with the Smartphone Addiction Scale (SAS) and occupational balance with the Adolescent Occupational Balance Scale (A-OBS). The A-OBS total score was the primary outcome, and its four dimensions were secondary outcomes. In the common complete-case sample (*n* = 1573), the crude inverse association between SAS and total A-OBS was markedly attenuated after district adjustment and was not statistically significant in the fully adjusted model (B = −0.027, 95% CI −0.057 to 0.003; β = −0.055; *p* = 0.078). Small inverse associations were observed for Rest and Sleep (β = −0.086), Occupational Variations and Adaptation (β = −0.099), and Occupational Meaning and Value (β = −0.071), but not for Temporality; all three significant domain associations remained below 0.05 after Holm correction. Sensitivity analyses produced broadly similar patterns. Overall, PSU was not robustly associated with total occupational balance after contextual adjustment, while selected dimensions showed small associations requiring cautious interpretation and longitudinal confirmation.

## 1. Introduction

Smartphones are deeply embedded in adolescents’ daily lives, and concerns have grown about use patterns that may interfere with their well-being and everyday functioning. Research on problematic smartphone use (PSU) has therefore expanded rapidly, although the construct remains heterogeneous in both terminology and measurement (Elhai et al., 2020; Harris et al., 2020; Montag et al., 2021). In adolescents, the broader digital-media literature suggests that associations with well-being and mental health are generally small, heterogeneous, and strongly context-dependent (Dienlin & Johannes, 2020; Odgers & Jensen, 2020; Orben et al., 2024). WHO surveillance has documented substantial levels of problematic social-media use and gaming-related problems among adolescents across Europe, Central Asia, and Canada (Boniel-Nissim et al., 2024). Systematic reviews and adolescent studies have further linked mobile and wireless-device use, as well as broader screen and social-media use, to mental-health symptoms and sleep problems (Girela-Serrano et al., 2024; Nagata et al., 2025), while smartphone-related problems have been associated with decreased social relationships (Alkın et al., 2020). Because “smartphone addiction” is not established here as a clinical diagnosis and the Smartphone Addiction Scale (SAS) was analyzed without a diagnostic cutoff, we use the descriptive term PSU throughout this study.

Most research on adolescent digital behavior has focused on mental-health, sleep, or physical-health outcomes, but smartphone use may also be relevant to how young people organize and experience their everyday occupations. Occupational balance is conceptually distinct from physical activity or sedentary behavior. More broadly, occupational balance has been described as an individual’s subjective perception of having the right amount and variety of occupations in daily life (Dhas & Wagman, 2022). This perspective captures the organization and subjective quality of everyday life rather than simply the amount of physical activity or screen exposure.

Smartphone-related habits may fragment routines, delay bedtime, or crowd out activities that would otherwise be restorative. Sleep is the most well-documented effect. Prospective studies and systematic reviews have linked interactive-device use and PSU to irregular sleep in adolescents (Dibben et al., 2023), and adolescent samples have shown specific associations with bedtime procrastination and reduced sleep quality (Acikgoz et al., 2022; Bozkurt et al., 2024). A 2025 umbrella review reached a similar conclusion for late-night and problematic device use, reporting delayed bedtimes and shorter sleep duration (Fiore et al., 2025). Prospective and cohort studies have extended this to later sleep problems, depression, anxiety, or related psychosocial difficulties (Carter et al., 2024; Chang et al., 2022). One adolescent randomized trial found that cutting back on problematic mobile-phone use was accompanied by improvements in depressive symptoms (Liu et al., 2024). Apart from the sleep literature, recent meta-analyses have reported lower physical activity and greater physical inactivity or sedentary behavior among adolescents and young adults who score higher on mobile-phone or smartphone addiction measures (Nambirajan et al., 2025; W. Xiao et al., 2022). PSU has also been associated with obesity in school-age samples (Ma et al., 2021) and with broader health indicators in university students (Zhang et al., 2024).

Evidence directly linking PSU to occupational balance remains limited. Studies in university students have reported associations between smartphone-related problems or problematic internet use and reduced occupational balance (Pekçetin et al., 2021; Romero-Tébar et al., 2021). For high school students, the evidence is even more current: A 2026 study using the A-OBS found reduced occupational balance in students with higher levels of nomophobia (Tekeci et al., 2026). Meanwhile, longitudinal work has found that individual, parental, and school factors all shape how PSU develops over time (Yoo, 2024), and Turkish adolescent studies have linked smartphone-related problems to social-relationship quality, loneliness, and depression (Sarman & Çiftci, 2024). Few studies have examined individual A-OBS dimensions while also accounting for school or district context, since digital behavior and everyday occupations may vary with the school a student attends, the neighborhood they live in, their family circumstances, and the academic demands they face.

Using the Smartphone Addiction Scale (SAS) and the Adolescent Occupational Balance Scale (A-OBS), we hypothesized an inverse association between PSU/SAS and total A-OBS scores among high school students. We examined the four A-OBS dimensions as secondary exploratory outcomes. And also, this study aims to inform future school-based interventions and public health strategies for adolescents by providing evidence-based insights into the behavioral patterns of young smartphone users.

## 2. Materials and Methods

### 2.1. Study Design and Participants

A cross-sectional school-based study was conducted between May and June 2024 in Sultanbeyli, Fatih, and Eyüpsultan, three districts of Istanbul, Türkiye. The districts were selected to capture differing sociodemographic contexts. Within each district, two public high schools were randomly selected from a list of public high schools in that district, yielding six participating schools. All students in the target population of the selected schools (*N* = 4895) were eligible for invitation; 2681 participated, for a participation rate of 54.7%. Because recruitment targeted the full eligible population of the selected schools, no separate a priori sample-size calculation was used for recruitment. Grade level (9th–12th) was recorded, but chronological age was not collected as a separate variable. Data were collected using self-administered questionnaires completed during class sessions.

### 2.2. Ethical Considerations

The Istanbul Medipol University Non-Interventional Clinical Research Ethics Committee approved the study (Decision No. 412, 18 April 2024), and it was conducted in accordance with the Declaration of Helsinki. Because the participants were high school students, written informed consent was obtained from a parent or legal guardian before participation. Consent forms were distributed to students through their classroom teachers, taken home for parent/legal-guardian signature, and returned to the school before data collection. Students were also informed about the purpose and procedures of the study; participation was voluntary, and questionnaire responses were kept confidential and used only for scientific purposes. Questionnaires were completed in the classroom setting.

### 2.3. Measures

#### 2.3.1. Sociodemographic and Behavioral Characteristics

The questionnaire recorded district, school, sex, grade level, maternal and paternal education and employment, mode of transportation to school, diagnosed illness, medication use, regular exercise, exercise frequency and duration, and average daily non-smartphone screen time on tablets, televisions, and computers. Regular exercise was a self-reported yes/no item; no minimum frequency or duration threshold was imposed, and days per week and typical session duration were recorded separately. The screen-time item did not measure smartphone-use duration. Smartphone ownership, daily smartphone-use duration, bedtime use, main smartphone activities, and social-media/gaming use were not collected separately.

#### 2.3.2. Smartphone Addiction Scale (SAS)

PSU was assessed with the 33-item Smartphone Addiction Scale (SAS), originally developed by Kwon et al. (Kwon et al., 2013) and adapted for Turkish high school students by Şata et al. (Şata et al., 2016). Each item is rated on a six-point Likert scale, producing a total score from 33 to 198. Higher scores indicate greater smartphone-related problems. No diagnostic cut-off was used; the SAS total score was analyzed as a continuous exposure. In the present data set, the SAS showed high internal consistency (Cronbach’s α = 0.940).

#### 2.3.3. Adolescent Occupational Balance Scale (A-OBS)

The Adolescent Occupational Balance Scale (A-OBS), developed by Güney Yılmaz et al. (Güney Yılmaz et al., 2023), assesses occupational balance. The scale contains 27 items rated on a four-point Likert scale and yields a total score ranging from 27 to 108, with higher scores indicating better perceived occupational balance. The official four dimensions are Temporality (items 1–15), Rest and Sleep (items 16–18), Occupational Variations and Adaptation (items 19–23), and Occupational Meaning and Value (items 24–27). Dimension scores were calculated by summing the corresponding items only when all constituent items were present and within the valid response range; partially completed scales were not prorated. Temporality comprises 15 of the 27 items (55.6% of total items), which is important to consider when interpreting the total score.

### 2.4. Statistical Analysis

Descriptive statistics were reported as numbers and percentages for categorical variables and as mean ± standard deviation for continuous variables. Group differences in SAS and A-OBS total scores were examined using independent-samples *t* tests or one-way ANOVA, as appropriate, with Cohen’s *d* and η^2^ reported as effect-size measures. The crude association between SAS and A-OBS total scores was assessed using Pearson correlation.

Multiple linear regression was used to examine the association between SAS scores and the A-OBS total score and its four dimensions. All regression models used the same complete-case sample (*n* = 1573). Models were adjusted for district, sex, grade, parental education and employment, and daily tablet/television/computer screen time. Covariates were selected a priori on conceptual grounds because of their plausible associations with both digital behavior and occupational patterns, rather than on the basis of univariate statistical significance. Because heteroskedasticity was present, HC3 robust standard errors were used. The four secondary A-OBS dimension analyses were adjusted for multiple testing using the Holm procedure.

Internal consistency was evaluated using Cronbach’s alpha and McDonald’s omega, and a four-factor CFA was conducted as a supportive psychometric analysis was performed for the A-OBS. Sensitivity analyses included school fixed-effects models, models with additional health and behavioral covariates, and inverse-probability weighting for complete-case inclusion. Regression diagnostics included variance inflation factors, a quadratic SAS term, the Breusch–Pagan test, residual distribution, and Cook’s distance. All tests were two-sided, with *p* < 0.05 considered statistically significant. Analyses were performed using IBM SPSS Statistics version 25.0 and Python 3.13.5 (NumPy 2.3.5, SciPy 1.17.0, and statsmodels 0.14.6).

## 3. Results

### 3.1. Participants and Missing Data

Of 4895 eligible students in the selected schools, 2681 participated (54.7%). Complete SAS scores were available for 1953 participants (72.8% of participants) and complete A-OBS total scores for 2095 (78.1%). Both total scales were complete for 1660 students (61.9%). After requiring complete data for the prespecified primary covariates, the common primary analytic sample comprised 1573 students (58.7% of participants). Domain completeness was higher for the shorter A-OBS dimensions: Temporality *n* = 2286, Rest and Sleep *n* = 2506, Occupational Variations and Adaptation *n* = 2454, and Occupational Meaning and Value *n* = 2482. However, all regression analyses across the four domains used the same primary analytic sample (*n* = 1573) (Table 1).

Complete-case inclusion was related most strongly to school (Cramér’s V = 0.350, *p* < 0.001) and district (Cramér’s V = 0.336, *p* < 0.001). Smaller differences were observed by sex, grade, maternal and paternal education, maternal employment, transportation, and screen-time category. Inclusion was not meaningfully associated with paternal employment, diagnosed illness, medication use, or regular exercise. School-specific complete-case proportions ranged from 35.2% to 77.9%, motivating the IPW sensitivity analysis (Supplementary Tables S1 and S2). The sociodemographic and other characteristics of the study participants, reported using variable-specific valid denominators, are presented in Table 2.

Unadjusted descriptive comparisons of SAS and A-OBS total scores across sociodemographic and behavioral characteristics are presented in Table 3 and Table 4. These comparisons describe unadjusted sample-level patterns; adjusted inferences are presented in the subsequent regression analyses.

### 3.2. A-OBS Psychometric Findings in the Present Sample

Internal consistency was high for the A-OBS total score and acceptable-to-high for all dimensions (Table 5). In the exploratory maximum-likelihood CFA, RMSEA (0.070) and SRMR (0.053) were within commonly used acceptable ranges, whereas CFI (0.883) and TLI (0.871) were below conventional 0.90 thresholds. This pattern was similar to the original validation study, which also reported CFI and TLI values below 0.90 (Güney Yılmaz et al., 2023). Because the present CFA treated four-category responses as approximately continuous rather than using an ordinal estimator, these fit indices are supportive descriptive evidence and should not be interpreted as definitive psychometric revalidation.

### 3.3. Primary Association with Overall Occupational Balance

Among the 1660 students with both total scales available, SAS and A-OBS total scores were modestly inversely correlated (r = −0.239, *p* < 0.001). In the common analytic sample (*n* = 1573), the crude standardized SAS coefficient was β = −0.248 (*p* < 0.001). Adding district reduced the coefficient to β = −0.049 (*p* = 0.117), indicating that most of the crude attenuation occurred after accounting for district context. Adding sex, grade, parental factors, and non-smartphone screen time changed the coefficient only minimally. In the final model, SAS was not statistically significantly associated with total A-OBS (B = −0.027, 95% CI −0.057 to 0.003; β = −0.055; *p* = 0.078). SAS added ΔR^2^ = 0.0025 beyond the covariates. A 10-point higher SAS score corresponded to an estimated 0.27-point lower A-OBS total score, and a one-SD higher SAS score corresponded to approximately a 0.93-point lower A-OBS total score, underscoring the small practical magnitude (Table 6).

### 3.4. Secondary A-OBS Dimension Analyses

Using the same *n* = 1573 analytic sample for all four dimensions, SAS was not associated with Temporality after adjustment. Small inverse associations were observed for Rest and Sleep, Occupational Variations and Adaptation, and Occupational Meaning and Value. All three remained below 0.05 after Holm correction for the four secondary tests (Table 7). These results indicate associations with selected dimensions but do not establish that the coefficients themselves are statistically different from one another.

### 3.5. Sensitivity Analyses

Sensitivity analyses yielded broadly similar estimates. Replacing district indicators with school fixed effects did not materially change the primary total-score association, and inverse-probability-weighted analyses addressing differential complete-case inclusion produced the same overall interpretation. Additional adjustment for diagnosed illness, medication use, regular exercise, and transportation also did not materially alter the findings (Supplementary Table S4).

## 4. Discussion

We conducted this research to test whether adolescents with higher PSU scores report reduced occupational balance, using a single analytic sample for the primary and dimension-level models, explicit correction for the secondary tests, and sensitivity analyses for school context and missingness. The crude inverse association between SAS and total A-OBS scores was markedly attenuated after district adjustment and was no longer statistically significant in the fully adjusted model. The three secondary dimensions that did reach significance produced small coefficients, and we read them as modest correlational findings rather than as evidence of a large or uniform effect. There is in any case little reason to expect uniformity: longitudinal work has identified distinct PSU trajectories across adolescence, with self-regulation, fear of missing out, and depressive symptoms distinguishing the higher-risk patterns (B. Xiao et al., 2025).

The marked attenuation after district adjustment also warrants consideration. District was strongly associated with both SAS and A-OBS scores and with complete-case inclusion. Accordingly, the attenuation after district adjustment should be interpreted cautiously because the same contextual factor was related to the exposure, the outcome, and who remained in the analytic sample. Although the IPW sensitivity analysis produced a similar pattern, it was based on measured covariates and therefore cannot fully exclude residual context-linked selection bias from unmeasured school- or district-level characteristics. District and school context may reflect differences in socioeconomic conditions, school routines, academic expectations, peer environments, access to digital technologies, and opportunities for occupations that do not involve a screen. Longitudinal evidence supports this view, showing that parental and school factors help shape trajectories of problematic smartphone use (Yoo, 2024), while studies of Turkish high school studies have linked smartphone-related problems to social-relationship quality and psychosocial context (Alkın et al., 2020; Sarman & Çiftci, 2024). A crude association between PSU and occupational balance may therefore reflect differences between schools and districts. A three-wave adolescent study is instructive here: it identified time-allocation and academic-stress pathways from PSU to later developmental outcomes, and while the time-allocation pathways remained significant after controlling for baseline outcomes, the academic-stress pathway was attenuated (Kim, 2026). Cross-sectional associations such as those reported here should therefore be interpreted in light of this broader contextual and temporal background.

The association observed for Rest and Sleep findings is consistent with previous findings in adolescents. Interactive electronic-device use has been prospectively linked to poor sleep and subsequent mental-health outcomes (Dibben et al., 2023). In Turkish adolescents, smartphone addiction scores track with sleep quality (Acikgoz et al., 2022), and Bozkurt et al. showed that bedtime procrastination mediates the relationship between PSU and sleep quality (Bozkurt et al., 2024). Fiore et al., synthesizing 127 primary studies, found that digital-device use—late-night and problematic patterns in particular—was associated with delayed bedtime, shorter sleep, and reduced sleep quality (Fiore et al., 2025). A longitudinal meta-analysis of 23 adolescent studies adds that dysfunctional digital-media use predicts subsequent sleep health, with some evidence that the relationship runs both ways (Pagano et al., 2023). However, the association in our study was small: a 10-point higher SAS score corresponded to only about 0.06 points lower on the 3–12 Rest and Sleep dimension after adjustment.

Of the four secondary dimensions, Occupational Variations and Adaptation showed the largest adjusted standardized coefficient, though the magnitude was still small (β = −0.099). This finding is consistent with previous occupational-balance studies in university and high school populations (Pekçetin et al., 2021; Romero-Tébar et al., 2021; Tekeci et al., 2026). Preoccupation with a smartphone may well coexist with a narrower or less adaptable repertoire of occupations. A cross-sectional design cannot determine the direction of the association—whether PSU constrains occupational variety, whether limited occupational opportunities drive students toward their phones, or whether a shared contextual factor produces both. Evidence from university students provides some support for this interpretation: digital life balance was positively related to occupational balance and inversely related to nomophobia (Aydemir et al., 2025), suggesting that how technology is woven into everyday roles may matter alongside how much of it there is. Similarly, a 2026 Turkish study found that social-media addiction was not an independent predictor of occupational balance after multivariable adjustment, whereas sleep quality remained associated with it (Yaman Dağdelen & Bahadır, 2026).

The association with Occupational Meaning and Value should also be interpreted cautiously. Tong et al. found longitudinal, bidirectional associations between meaning in life and problematic mobile-phone use in adolescents (Tong et al., 2026)—evidence that argues directly against assuming smartphone use comes first, and a diminished sense of meaning follows. Our cross-sectional estimate is equally consistent with reciprocal processes or with shared determinants, and it should be described as an association, not an effect.

Temporality showed no adjusted association. This has implications for the total score, because Temporality supplies 15 of the 27 items and so contributes 55.6% of the unweighted total; the total is therefore weighted more heavily toward this one dimension than toward the three shorter ones. That structural feature may help explain why the overall association was weak, even though three of the four dimensions produced small associations. One caveat, though: significance in some dimensions and not in others does not establish that the underlying coefficients differ, and we did not base our conclusions on formal between-coefficient comparisons.

The small effect sizes also limit the practical significance of these findings. The largest secondary standardized coefficient was about −0.10, and the additional variance attributable to SAS ranged from roughly 0.01% for Temporality to 0.80% for Occupational Variations and Adaptation. Recent studies suggest a similar pattern: the links between digital-technology use and adolescent well-being are generally small and can vary across individuals and settings (Odgers & Jensen, 2020; Orben et al., 2024). With a sample of this size, statistical significance says little about whether an effect is clinically, educationally, or functionally meaningful.

Findings from the broader health literature provide additional context, although these outcomes are distinct from occupational balance. Meta-analyses indicate that greater mobile-phone addiction is associated with lower physical activity (W. Xiao et al., 2022) and more sedentary behavior (Nambirajan et al., 2025). School-age research has linked PSU with obesity (Ma et al., 2021), while research in university students has reported associations between smartphone addiction and broader health indicators (Zhang et al., 2024). Turkish adolescent data also link smartphone addiction with loneliness and depression (Sarman & Çiftci, 2024). These findings support concern about problematic digital behavior, but they do not measure occupational balance, which concerns the perceived pattern, restoration, adaptability, and meaning of everyday occupations as captured by the A-OBS.

The unadjusted comparisons by sex, grade, parental characteristics, transportation, and screen-time category in Table 3 and Table 4 are reported for descriptive purposes only; we do not interpret them as independent predictors. District differences were large, and demographic comparisons in a sample like this are easily confounded by school and contextual composition. Our discussion accordingly rests on the adjusted relationship between PSU and occupational balance rather than on those unadjusted comparisons.

### Limitations

This study has several limitations. First, the cross-sectional design rules out causal or temporal inference; reciprocal relationships are entirely plausible. Second, every measure was self-reported and therefore open to recall and social-desirability bias. Third, missingness was substantial. Only 58.7% of participants entered the primary complete-case model, and inclusion varied by district and school. District was also strongly associated with both SAS and A-OBS scores, meaning that the same contextual factor was related to the exposure, the outcome, and who remained in the analytic sample. Although IPW sensitivity analyses gave similar estimates, IPW relied on measured covariates and cannot fully rule out residual context-linked selection bias from unmeasured district- or school-level characteristics. Fourth, the sample came from six public schools in three selected Istanbul districts, so the results should not be generalized to adolescents in Istanbul or Türkiye as a whole. Fifth, we did not collect chronological age separately. Since the original A-OBS development study was conducted in healthy adolescents aged 12–16 years (Güney Yılmaz et al., 2023), we cannot confirm that every participant, particularly those in the upper grades, fell within the validation age range. Sixth, 19.5% of participants reported a diagnosed illness, and we did not have detailed diagnostic information for this variable. Seventh, we did not measure smartphone ownership, smartphone-use duration, bedtime use, specific applications, social-media and gaming activity, or the timing and context of use; the tablet, television, and computer screen-time variable is not a proxy for smartphone exposure and should not be read as one. Eighth, the supportive CFA treated the four-category A-OBS items as approximately continuous and used maximum-likelihood estimation rather than an ordinal estimator such as WLSMV, so the fit indices warrant caution. Ninth, school fixed effects capture between-school heterogeneity but leave residual within-school correlation unaddressed, and with only six schools the scope for cluster-based inference is limited. Tenth, although written parental/legal-guardian consent was obtained and students were informed that participation was voluntary, a separate written adolescent assent form was not used; this should be considered an ethical limitation. Finally, residual confounding remains possible—by household socioeconomic resources, parental monitoring, academic demands, peer context, and other school-level characteristics we did not measure.

## 5. Conclusions

In this six-school sample, the crude inverse association between PSU and overall occupational balance was largely attenuated after accounting for district context and was not statistically significant in the fully adjusted primary model. Secondary analyses identified small inverse associations with Rest and Sleep, Occupational Variations and Adaptation, and Occupational Meaning and Value, but not Temporality. Rest and Sleep and Occupational Variations and Adaptation were the most robust secondary findings across multiplicity-adjusted sensitivity analyses, whereas the Meaning and Value association was less stable after correction in some sensitivity models. Overall, coefficient directions and magnitudes were broadly similar across sensitivity analyses. Interpret the findings as modest, cross-sectional associations rather than evidence of a clinical smartphone addiction syndrome, domain-specific causality, or intervention effects. Longitudinal, school-aware studies that characterize smartphone-use timing, content, and context are needed to clarify directionality and practical significance.

## Figures and Tables

**Table 1:** Analytical sample flow and scale completeness.

Stage/Score	*n*	Percentage
Eligible target population in selected schools	4895	100.0% of eligible
Participated	2681	54.7% of eligible
Did not participate	2214	45.3% of eligible
Complete SAS total	1953	72.8% of participants
Complete A-OBS total	2095	78.1% of participants
Both SAS and A-OBS total complete	1660	61.9% of participants
Primary common complete-case analytic sample	1573	58.7% of participants
A-OBS Temporality complete	2286	85.3% of participants
A-OBS Rest and Sleep complete	2506	93.5% of participants
A-OBS Occupational Variations and Adaptation complete	2454	91.5% of participants
A-OBS Occupational Meaning and Value complete	2482	92.6% of participants

For the first three rows, percentages use the eligible target population (*N* = 4895) as the denominator; subsequent completeness percentages use the 2681 participating students as the denominator. Scale scores were calculated only when all items contributing to that total or dimension were present and within the valid response range.

**Table 2:** Participant characteristics with variable-specific valid denominators.

Characteristic	Category	*n*	%	Valid *N*
District	Sultanbeyli	1275	47.6	2681
	Fatih	399	14.9	2681
	Eyüpsultan	1007	37.6	2681
Sex	Female	1770	66.3	2671
	Male	901	33.7	2671
Grade	9th	954	35.7	2674
	10th	843	31.5	2674
	11th	665	24.9	2674
	12th	212	7.9	2674
Maternal education	Illiterate	202	7.6	2642
	Literate/no formal education	181	6.9	2642
	Primary school	908	34.4	2642
	Middle school	607	23.0	2642
	High school	571	21.6	2642
	University	173	6.5	2642
Maternal employment	Not employed	1808	69.5	2602
	Employed	794	30.5	2602
Paternal education	Illiterate	67	2.5	2635
	Literate/no formal education	127	4.8	2635
	Primary school	809	30.7	2635
	Middle school	685	26.0	2635
	High school	717	27.2	2635
	University	230	8.7	2635
Paternal employment	Not employed	202	7.7	2628
	Employed	2426	92.3	2628
Daily tablet/TV/computer screen time	<30 min	224	8.4	2662
	30 min–2 h	591	22.2	2662
	2–4 h	920	34.6	2662
	>4 h	927	34.8	2662
Diagnosed illness	No	2106	80.5	2617
	Yes	511	19.5	2617
Medication use	No	2246	85.4	2630
	Yes	384	14.6	2630
Regular exercise	No	1636	62.3	2624
	Yes	988	37.7	2624
Mode of transportation to school	Car	376	14.1	2666
	School bus	277	10.4	2666
	Walking	894	33.5	2666
	Bicycle	23	0.9	2666
	Other	1096	41.1	2666

Percentages use the valid denominator for each characteristic. Regular exercise was self-defined rather than threshold-based. Among students reporting regular exercise who also provided positive follow-up values, the median frequency was 4 days/week (IQR 3–5; *n* = 742) and the median session duration was 60 min (IQR 30–90; *n* = 727).

**Table 3:** Comparison of SAS total scores according to sociodemographic and behavioral characteristics.

Characteristic	Category	*n*	Mean ± SD	t/F	*p*	Effect Size
District	Sultanbeyli	767	97.1 ± 33.3	F = 197.616	<0.001	η^2^ = 0.169
	Fatih	306	99.5 ± 30.4			
	Eyüpsultan	880	126.0 ± 29.7			
Sex	Female	1320	111.7 ± 33.3	t = 2.283	0.023	d = 0.111
	Male	629	108.0 ± 36.2			
Grade	9th	684	109.1 ± 32.9	F = 15.527	<0.001	η^2^ = 0.023
	10th	645	111.8 ± 35.3			
	11th	480	115.8 ± 33.4			
	12th	139	94.1 ± 33.6			
Maternal employment	Not employed	1282	108.1 ± 34.6	t = −4.957	<0.001	d = −0.241
	Employed	628	116.3 ± 32.4			
Maternal education	Illiterate	132	103.8 ± 33.1	F = 5.000	<0.001	η^2^ = 0.013
	Literate/no formal education	121	103.0 ± 36.0			
	Primary school	651	108.4 ± 32.7			
	Middle school	429	111.4 ± 35.2			
	High school	455	115.1 ± 34.1			
	University	142	115.5 ± 36.3			
Paternal education	Illiterate	36	103.5 ± 29.6	F = 3.767	0.002	η^2^ = 0.010
	Literate/no formal education	76	99.0 ± 34.9			
	Primary school	584	107.8 ± 33.4			
	Middle school	488	111.6 ± 33.7			
	High school	561	112.8 ± 35.1			
	University	178	114.2 ± 36.2			
Paternal employment	Not employed	142	107.3 ± 29.5	t = −1.210	0.226	d = −0.106
	Employed	1776	110.9 ± 34.6			
Transportation	Car	251	102.1 ± 33.2	F = 5.266	<0.001	η^2^ = 0.011
	School bus	193	110.8 ± 36.8			
	Walking	677	112.9 ± 33.6			
	Bicycle	18	100.3 ± 33.0			
	Other	805	111.5 ± 34.0			
Screen time	<30 min	144	98.3 ± 40.1	F = 14.194	<0.001	η^2^ = 0.022
	30 min–2 h	416	104.8 ± 37.4			
	2–4 h	675	112.6 ± 33.4			
	>4 h	706	114.5 ± 30.5			
Diagnosed illness	No	1542	110.1 ± 34.1	t = −1.054	0.292	d = −0.060
	Yes	383	112.2 ± 34.8			
Medication use	No	1644	109.9 ± 34.0	t = −1.670	0.095	d = −0.108
	Yes	283	113.6 ± 35.0			
Regular exercise	No	1203	110.4 ± 33.0	t = −0.339	0.735	d = −0.016
	Yes	720	110.9 ± 36.0			

Values are mean ± SD among participants with a complete score for the relevant scale. Tests are unadjusted and descriptive. Cohen’s d is reported for two-group comparisons and η^2^ for one-way ANOVA comparisons. These analyses are not interpreted as independent predictors.

**Table 4:** Comparison of A-OBS total scores according to sociodemographic and behavioral characteristics.

Characteristic	Category	*n*	Mean ± SD	t/F	*p*	Effect Size
District	Sultanbeyli	871	73.9 ± 15.8	F = 306.695	<0.001	η^2^ = 0.227
	Fatih	319	72.4 ± 16.6			
	Eyüpsultan	905	57.2 ± 13.3			
Sex	Female	1423	66.0 ± 15.9	t = −1.580	0.114	d = −0.074
	Male	669	67.3 ± 19.0			
Grade	9th	733	66.5 ± 16.5	F = 12.709	<0.001	η^2^ = 0.018
	10th	682	67.2 ± 17.1			
	11th	517	63.4 ± 17.1			
	12th	159	72.4 ± 16.0			
Maternal employment	Not employed	1387	67.4 ± 16.9	t = 4.615	<0.001	d = 0.219
	Employed	655	63.8 ± 16.7			
Maternal education	Illiterate	157	68.4 ± 16.6	F = 6.810	<0.001	η^2^ = 0.016
	Literate/no formal education	128	71.2 ± 14.9			
	Primary school	695	67.6 ± 16.4			
	Middle school	458	66.1 ± 17.6			
	High school	482	63.2 ± 16.9			
	University	151	65.3 ± 17.5			
Paternal education	Illiterate	51	65.8 ± 18.1	F = 2.361	0.038	η^2^ = 0.006
	Literate/no formal education	78	69.8 ± 14.8			
	Primary school	613	67.9 ± 16.4			
	Middle school	527	66.0 ± 16.8			
	High school	601	65.4 ± 17.3			
	University	192	65.1 ± 17.7			
Paternal employment	Not employed	158	65.1 ± 15.4	t = −1.003	0.316	d = −0.083
	Employed	1900	66.5 ± 17.1			
Transportation	Car	295	67.5 ± 17.6	F = 0.849	0.494	η^2^ = 0.002
	School bus	199	64.8 ± 16.6			
	Walking	726	66.6 ± 16.3			
	Bicycle	16	68.0 ± 19.8			
	Other	849	66.2 ± 17.3			
Screen time	<30 min	172	67.6 ± 16.7	F = 5.353	0.001	η^2^ = 0.008
	30 min–2 h	443	68.3 ± 17.0			
	2–4 h	727	66.8 ± 17.2			
	>4 h	739	64.6 ± 16.5			
Diagnosed illness	No	1655	66.6 ± 17.2	t = 0.507	0.612	d = 0.028
	Yes	404	66.1 ± 16.0			
Medication use	No	1764	66.7 ± 16.9	t = 1.322	0.186	d = 0.083
	Yes	300	65.3 ± 17.4			
Regular exercise	No	1296	66.4 ± 15.2	t = −0.183	0.855	d = −0.008
	Yes	762	66.5 ± 19.6			

Values are mean ± SD among participants with a complete score for the relevant scale. Tests are unadjusted and descriptive. Cohen’s d is reported for two-group comparisons and η^2^ for one-way ANOVA comparisons. These analyses are not interpreted as independent predictors.

**Table 5:** Internal consistency of SAS and A-OBS scores.

Scale/Dimension	Complete *n*	Cronbach α	McDonald ω
SAS total	1953	0.940	0.940
A-OBS total	2095	0.944	0.950
Temporality	2286	0.897	0.902
Rest and Sleep	2506	0.811	0.833
Occupational Variations and Adaptation	2454	0.821	0.835
Occupational Meaning and Value	2482	0.808	0.823

Exploratory four-factor A-OBS CFA on complete A-OBS items (*n* = 2095; maximum-likelihood estimation treating four-category items as approximately continuous): χ^2^ = 3551.5, df = 318, CFI = 0.883, TLI = 0.871, RMSEA = 0.070, SRMR = 0.053.

**Table 6:** Sequential models for SAS and A-OBS total score (common analytic sample *n* = 1573).

Model	Covariates Added	B	Standardized β	95% CI	*p*	Adjusted R^2^
1	SAS only	−0.123	−0.248	−0.153 to −0.093	<0.001	0.061
2	+District	−0.024	−0.049	−0.054 to 0.006	0.117	0.265
3	+Sex + grade	−0.025	−0.051	−0.055 to 0.005	0.097	0.266
4	+Parental education/employment	−0.026	−0.053	−0.056 to 0.004	0.087	0.266
5	+Tablet/TV/computer screen time	−0.027	−0.055	−0.057 to 0.003	0.078	0.265

HC3 heteroskedasticity-robust standard errors were used. The final model R^2^ was 0.276 (adjusted R^2^ 0.265); SAS contributed ΔR^2^ = 0.0025 beyond covariates.

**Table 7:** Adjusted secondary associations between SAS and A-OBS dimensions (*n* = 1573 for all models).

A-OBS Dimension	B	Std. β	95% CI	Raw *p*	Holm *p*	Adj. R^2^	ΔR^2^ SAS	10-Point SAS Effect	1-SD SAS Effect
Temporality	−0.0036	−0.013	−0.020 to 0.013	0.665	0.665	0.314	0.0001	−0.036	−0.124
Rest and Sleep	−0.0064	−0.086	−0.0109 to −0.0019	0.006	0.017	0.089	0.0060	−0.064	−0.219
Occupational Variations and Adaptation	−0.0107	−0.099	−0.0177 to −0.0037	0.003	0.010	0.083	0.0080	−0.107	−0.368
Occupational Meaning and Value	−0.0064	−0.071	−0.0118 to −0.0011	0.018	0.035	0.249	0.0041	−0.064	−0.221

All models adjust for district, sex, grade, maternal and paternal education/employment, and daily tablet/TV/computer screen time, with HC3 robust inference. Domain analyses were secondary/exploratory; Holm adjustment was applied across the four domain tests.

## Data Availability

The data supporting the findings of this study are available from the corresponding author upon reasonable request, subject to ethical and institutional restrictions.

## Supplementary Materials

The following supporting information can be downloaded at https://www.mdpi.com/article/10.3390/bs16101855/s1. Table S1, Characteristics associated with inclusion in the primary complete-case analysis; Table S2, School-specific inclusion in the primary complete-case analysis; Table S3, Full coefficients from the primary adjusted linear regression model for total A-OBS (*n* = 1573); Table S4, Sensitivity analyses: standardized SAS coefficients (β). For the total A-OBS outcome, parentheses show the model *p* value; for the four secondary domains, parentheses show Holm-adjusted *p* values across the four domain tests.
