# Supplementary material for: Problematic Smartphone Use and Occupational Balance Among High School Students: A Cross-Sectional Study
Source: Behav Sci (Basel). 2026 Oct 9;16(10):1855. doi: 10.3390/bs16101855 (PMC13701229; doi:10.3390/bs16101855)
Supplement: Supplementary file 1 [file behavsci-16-01855-s001.zip › behavsci-4556325-supplementary.pdf]

Supplementary Tables

Supplementary Table S1. Characteristics associated with inclusion in the primary complete-case analysis

Characteristic	p value	Cramér's V	Interpretation
District	<0.001	0.336	Largest difference
Sex	<0.001	0.073	Small
Grade	0.002	0.076	Small
Maternal education	<0.001	0.134	Small-to-moderate
Maternal employment	<0.001	0.079	Small
Paternal education	<0.001	0.121	Small-to-moderate
Paternal employment	0.664	0.008	Negligible
Tablet/TV/computer screen time	0.001	0.077	Small
Transportation	0.013	0.069	Small
Diagnosed illness	0.647	0.009	Negligible
Medication use	0.806	0.005	Negligible
Regular exercise	0.208	0.025	Negligible
School	<0.001	0.350	Largest difference

Primary complete-case n = 1,573; excluded n = 1,108. School-specific complete-case proportions ranged from 35.2% to 77.9%.

Supplementary Table S2. School-specific inclusion in the primary complete-case analysis

School	Total n	Complete-case n	Complete-case %
Göktürk Ali Rıza Özderici A.L.	518	403	77.8
Haydar Akçelik	489	381	77.9
Kadırga Mesleki ve Teknik Anadolu Lisesi	230	131	57.0
Sultan Alparslan MTAL	542	191	35.2
Sultanbeyli Kız AİHL	733	345	47.1
Yedikule Anadolu Lisesi	169	122	72.2

Supplementary Table S3. Full coefficients from the primary adjusted linear regression model for total A-OBS (n = 1,573)

Predictor	B	95% CI	p
SAS total	-0.027	-0.057 to 0.003	0.078
District: Fatih vs Sultanbeyli	-1.508	-4.086 to 1.069	0.251
District: Eyüpsultan vs Sultanbeyli	-17.615	-19.594 to -15.636	<0.001
Sex: Female vs Male	0.468	-1.211 to 2.147	0.585
Grade: 10th vs 9th	1.053	-0.684 to 2.790	0.235
Grade: 11th vs 9th	0.373	-1.567 to 2.313	0.706
Grade: 12th vs 9th	-1.794	-5.303 to 1.715	0.316
Maternal education: Illiterate vs Primary	-0.016	-3.541 to 3.509	0.993
Maternal education: Literate/no formal vs Primary	2.619	-0.712 to 5.951	0.123
Maternal education: Middle vs Primary	0.272	-1.785 to 2.330	0.795
Maternal education: High school vs Primary	-0.814	-2.884 to 1.255	0.441
Maternal education: University vs Primary	1.529	-1.800 to 4.859	0.368

Maternal employment: Employed vs Not employed	0.359	-1.245 to 1.964	0.661
Paternal education: Illiterate vs Primary	-2.348	-10.604 to 5.907	0.577
Paternal education: Literate/no formal vs Primary	-4.774	-9.300 to -0.249	0.039
Paternal education: Middle vs Primary	0.243	-1.719 to 2.206	0.808
Paternal education: High school vs Primary	-0.413	-2.358 to 1.531	0.677
Paternal education: University vs Primary	-1.782	-4.796 to 1.232	0.247
Paternal employment: Not employed vs Employed	-2.420	-5.495 to 0.654	0.123
Screen time: 30 min–2 h vs <30 min	1.640	-1.763 to 5.043	0.345
Screen time: 2–4 h vs <30 min	1.620	-1.658 to 4.897	0.333
Screen time: >4 h vs <30 min	1.188	-2.139 to 4.515	0.484

HC3 heteroskedasticity-robust 95% confidence intervals and p values are shown. Reference categories are stated in the predictor labels. The model adjusted R^2 was 0.265; SAS contributed $\Delta R^2 = 0.0025$ beyond covariates.

Supplementary Table S4. Sensitivity analyses: standardized SAS coefficients (β). For the total A-OBS outcome, parentheses show the model p value; for the four secondary domains, parentheses show Holm-adjusted p values across the four domain tests.

**Analysis**	**Total A-OBS**	**Temporality**	**Rest and Sleep**	**Occupational Variations and Adaptation**	**Occupational Meaning and Value**
School fixed effects	-0.046 (p=0.139)	-0.005 (Holm p=0.871)	-0.079 (Holm p=0.033)	-0.089 (Holm p=0.027)	-0.064 (Holm p=0.063)
Extended covariates (n=1,537)	-0.059 (p=0.059)	-0.017 (Holm p=0.575)	-0.090 (Holm p=0.013)	-0.104 (Holm p=0.008)	-0.074 (Holm p=0.029)
IPW missing-data sensitivity	-0.053 (p=0.108)	-0.014 (Holm p=0.650)	-0.080 (Holm p=0.041)	-0.095 (Holm p=0.022)	-0.068 (Holm p=0.065)

Sensitivity analyses are presented as robustness checks rather than as additional primary hypothesis tests. The direction and magnitude of the estimates were broadly similar to the primary analysis; after Holm adjustment, the Occupational Meaning and Value association was less stable in the school-fixed-effects and IPW analyses.
